# Supplementary material for: Direct observation of the cell-wall remodeling in adhering Staphylococcus aureus 27217: An AFM study supported by SEM and TEM
Source: Cell Surf. 2019 Mar 5;5:100018. doi: 10.1016/j.tcsw.2019.100018 (PMC7389151; doi:10.1016/j.tcsw.2019.100018)
Supplement: Supplementary data 1 [file mmc1.pdf]

# Supplementary Information

## Figure captions

Figure SI1:

Principle of automatic detection of the "pixel-ratio" for each bacterium of the samples by a home-made Matlab program[1] constituted by the following steps:

1. the initial grayscale SEM image is converted to a binary one;
2. an on-screen first determination of the mean radius, $r_0$, of bacteria is performed;
3. the connected components in the binary image are detected and their edges determined;
4. these edges are approximated by circles with a variable radius the initial value of which was equal to the $r_0$, by using a circular Hough transform[2,3]. We checked the validity of such a process by verifying that all the cells were detected and that their final radius was in the range $r_0 \pm 10\%$;
5. successive dilation, erosion operations to increase the contrast of the cell surface structures for an automatic detection are done;
6. finally, the software automatically counts the ratio of the 'on' pixels, where these surface structures are were revealed, to the total number of pixels for each bacterium, the so-called "pixel-ratio".

Figure SI2:

SEM observation of *S. aureus* ATCC 27217 bacterial strain. Evidence of two types of self-adhering subpopulations: the so-called "*bald*" (figures SI2.b, SI2.d) and "*hairy*" cells (figures SI2.a, SI2.c). Figures SI2.c and SI2.d are numerical enlargements (with enhanced contrast) of images SI2.a and SI2.b (respectively) along the green squares. Scale bars: 500nm.

## References


1. Marlière, C. Matlab code available upon request : christian.marliere@u-psud.fr. (2018).
2. Yuen, H., Princen, J., Illingworth, J. & Kittler, J. Comparative study of Hough Transform methods for circle finding. *Image Vis. Comput.* **8**, 71–77 (1990).
3. Davies, E. R. *Machine Vision: Theory, Algorithms, Practicalities.* (Elsevier, 2012). doi:10.1016/C2010-0-66926-4

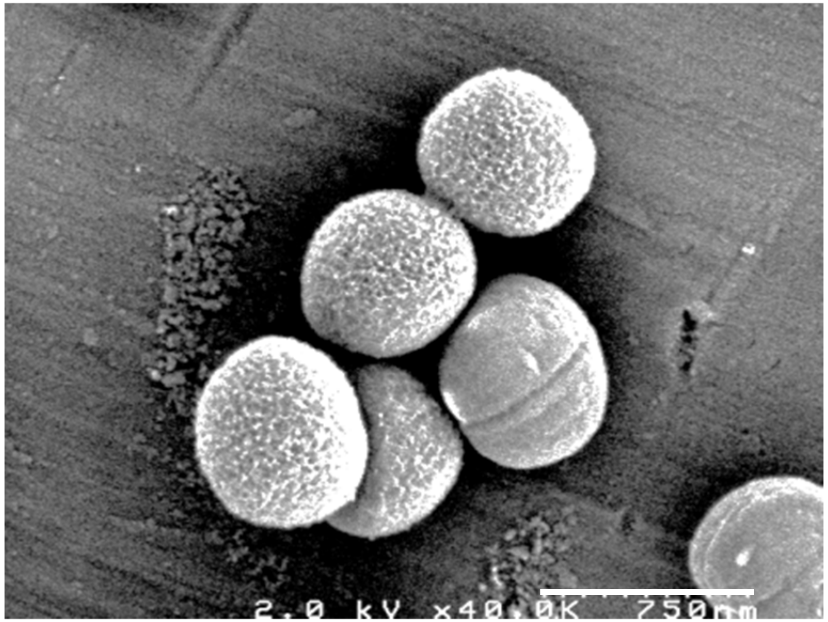


Figure SI1.a

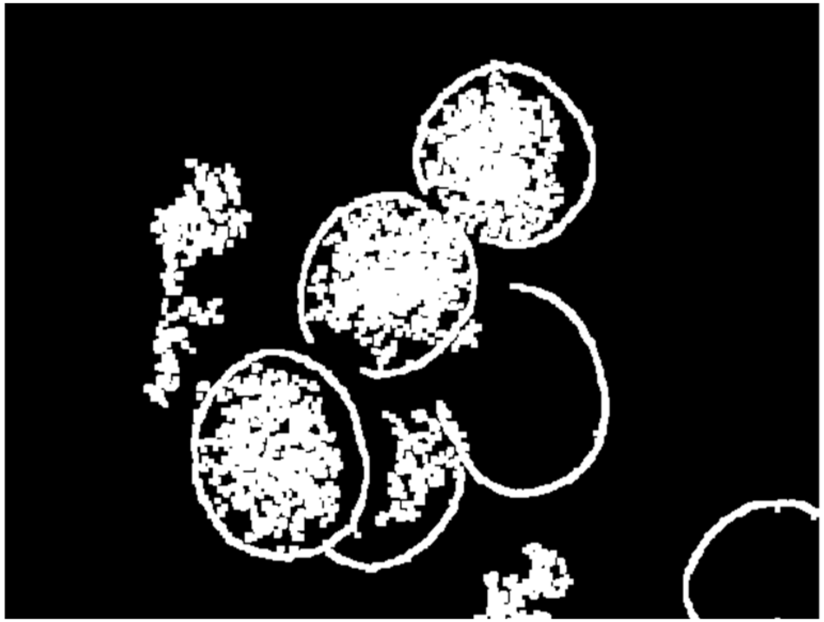

Figure SI1.b

Non- Centrifuged

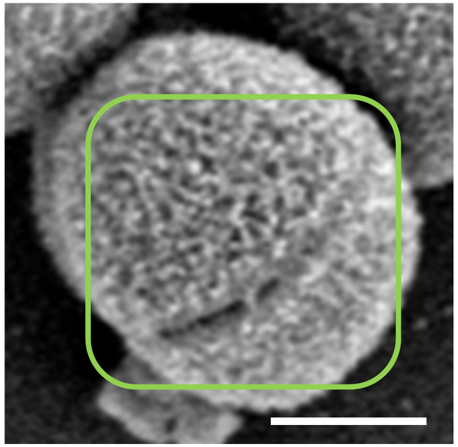

Figure SI2.a

Centrifuged

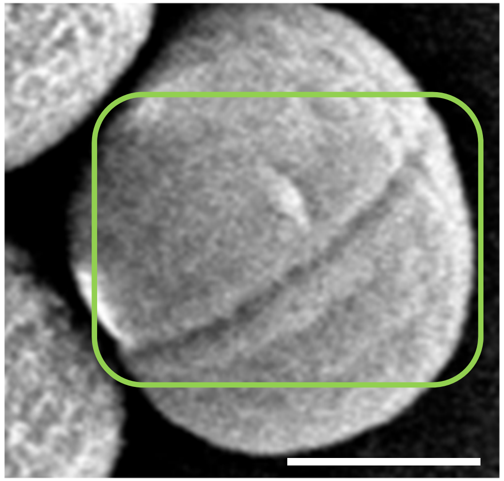

Figure SI2.b

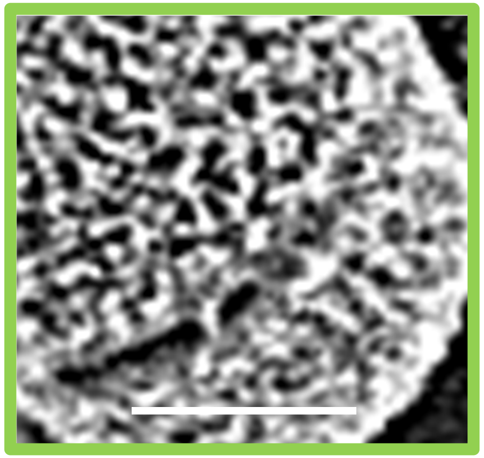

Figure SI2.c

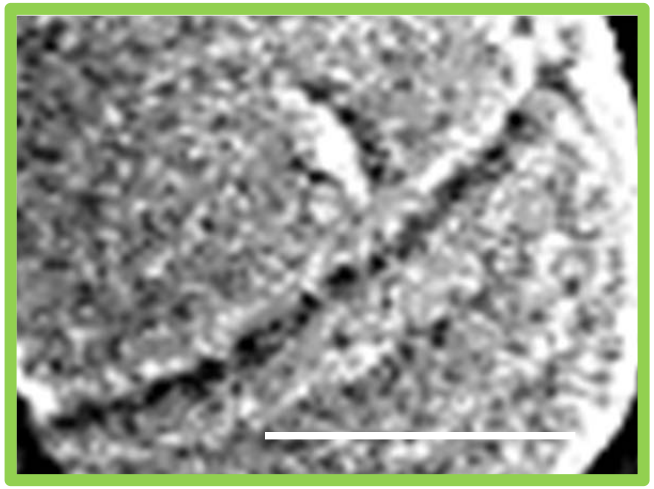

Figure SI2.d
